# What ethical approaches are used by scientists when sharing health data? An interview study

**DOI:** 10.1186/s12910-022-00779-8

**Published:** 2022-04-11

**Authors:** Jennifer Viberg Johansson, Heidi Beate Bentzen, Deborah Mascalzoni

**Affiliations:** 1 Centre for Research Ethics and Bioethics, Department of Public Health and Caring Sciences, Uppsala University, Box 564, 751 22 Uppsala, Sweden; 2 Institute for Futures Studies, Stockholm, Sweden; 3 Norwegian Research Center for Computers and Law, Faculty of Law, University of Oslo, Oslo, Norway; 4 Institute of Biomedicine, Eurac Research, Bolzano, Italy

**Keywords:** Information technology, Reusing and sharing health-related data, Scientists’ views, Ethical approaches

## Abstract

**Background:**

Health data-driven activities have become central in diverse fields (research, AI development, wearables, etc.), and new ethical challenges have arisen with regards to privacy, integrity, and appropriateness of use. To ensure the protection of individuals’ fundamental rights and freedoms in a changing environment, including their right to the protection of personal data, we aim to identify the ethical approaches adopted by scientists during intensive data exploitation when collecting, using, or sharing peoples’ health data.

**Methods:**

Twelve scientists who were collecting, using, or sharing health data in different contexts in Sweden, were interviewed. We used systematic expert interviews to access these scientists’ specialist knowledge, and analysed the interviews with thematic analysis. Phrases, sentences, or paragraphs through which ethical values and norms were expressed, were identified and coded. Codes that reflected similar concepts were grouped, subcategories were formulated, and categories were connected to traditional ethical approaches.

**Results:**

Through several examples, the respondents expressed four different ethical approaches, which formed the main conceptual categories: consideration of consequences, respect for rights, procedural compliance, and being professional.

**Conclusions:**

To a large extent, the scientists’ ethical approaches were consistent with ethical and legal principles. Data sharing was considered important and worth pursuing, even though it is difficult. An awareness of the complex issues involved in data sharing was reflected from different perspectives, and the respondents commonly perceived a general lack of practical procedures that would by default ensure ethical and legally compliant data collection and sharing. We suggest that it is an opportune time to move on from policy discussions to practical technological ethics-by-design solutions that integrate these principles into practice.

**Supplementary Information:**

The online version contains supplementary material available at 10.1186/s12910-022-00779-8.

## Background

New data-driven technologies are changing our societies, and the extensive and intensive use of health data can lead to positive outcomes in medicine and applications beneficial to our health. The rapid development of artificial intelligence and machine learning techniques brings improved abilities to find patterns in big data [1] that can facilitate new discoveries in health care, new diagnostic tools, and better treatments and help reserve resources for the welfare sector. Different actors, including technological and pharmaceutical companies, medical research institutions, healthcare providers and public health authorities, are collecting, using and increasingly sharing people’s individual-level health information (e.g., age, perceived health, disease group, and income) [2, 3]. The wide scope of big data use encompasses a broad range of information, including not only smartphone and wearable application data, medical data from healthcare providers, and research data but also social and demographic data, such as individual-level health data, that are used for different purposes or merged, aggregated and linked to optimize products and services in our society.

However, data sharing and the reuse of people’s data entail risks and challenges for people’s privacy, the fair use of data, and justice [4–6]. It is becoming increasingly difficult to apply old safeguards and oversight practices in data-intensive contexts where the very notion of consent and anonymization have changed [7, 8].

In the developing field of big data, ensuring the adequate protection of data subjects and communities while reaping the full benefits that new data-driven technology brings to our society presents a dilemma. There is a need to develop solutions and common practices for handling people’s data, from both the perspective of data subjects and that of collectors and legal entities, which may hold conflicting interests [9]. Despite the recent adoption of the EU General Data Protection Regulation (GDPR) and the proposal for an EU Artificial Intelligence Act [10], many procedures on how data use and data sharing should be governed responsibly are not practically in place [11].

Hence, the different domains that are dependent on data collected from the public must maintain public trust in relation to the collection and use of data. There is a growing body of literature about the preferences and attitudes of the public, research participants, and patients in relation to data sharing. Different factors contribute to people’s willingness to share data. Factors such as the level of identifiability, whether an overview of sharing practices is provided and the extent to which participants are informed are central elements that play an important role in people’s privacy concerns [12–16].

Discourses on how to govern health data extensively consider individuals’ security concerns; governance mechanisms for the collection, use and sharing of health data must meet social expectations regarding security and privacy, and enable broad use of health data for a variety of purposes that are beneficial to society. Scientists are one of the most significant stakeholder groups because they process individual-level health data. In doing so they must consider data sharing preferences and legal requirements and translate them into practice. It is therefore pertinent to explore scientists’ views and reasoning in regard to data use, and determine to what extent they align with current legal and ethical frameworks. Investigating the views of scientists who handle health data concerning how and why it is shared with different organizations can help identify areas that should be improved to meet various ethical and legal standards (e.g., GDPR). Moreover, identifying any discrepancies between what the scientist say they should do and what they say occurs in practice, can provide an insight into governance requirements.

The aim of this study was to explore scientists’ ethical reasoning and identify the ethical approaches attributed to scientists’ expressions about when they use and share health data. We believe that the scientists’ expressed experience and decision-making frameworks reflect their understanding of the ethical issue. Identifying the ethical approaches and reasoning behind data sharing decisions can provide insights regarding the requirements of new technical and legal solutions for the reuse of health data. In our civil society there is an expectation that ethical norms will become legal norms. Understanding where gaps exist between expectations towards data sharing, written law and ethically underpinned data sharing in practice may point to potential solutions for closing the gap to protect individuals’ rights.

## Method

This study is a secondary analysis of interviews of scientists who use health data, and it followed a study that examined health data users’ views on governance mechanisms that exist and that should be in place when health data are shared for new purposes with other actors [16, 17]. The focus of that primary analysis was on procedural issues and practical experiences related to sharing health data in the context of governance mechanisms.

Due to the richness of the material collected in Sweden and considering that the respondents extensively expressed the ethical dimension of sharing subjects’ data, secondary analysis [18] was performed to scrutinize interests independently from the original analysis.

### Design and theoretical framework

Empirical results can be helpful in terms of identifying problems of a moral and ethical nature and proving context sensitivity [19]. We argue that by identifying experts’ ethical approaches in the data-sharing field, we can unpack some of their embedded beliefs and attitudes towards data management. In turn, this information can inform our understanding of the issues faced in relation to data use and data sharing and thus reveal potential solutions. This article focuses on the identification of the ethical approaches expressed by scientists through their statements with regard to their experiences with collecting, using, and sharing health information, as how people’s health data should be handled not only is a practical issue but also often involves normative issues.

Scientists are facing ethical decisions and that put their moral compass and values into question. There are several traditional ethical approaches to consider when making decisions. Actions can be guided as right or wrong (judging according to conforming rules or duties) or good or bad (evaluating the consequences). Another ethical tradition focuses on the intention and the character of the moral agent (virtue ethics). Instead of focusing on the right act, that tradition focuses on how to be a certain sort of person [20]. We conducted systematic expert interviews with the aim of accessing knowledge and specialized information acquired by experts in specific fields [21].

### Respondents

We invited scientists in the field of health data use to describe how data-driven activities are conducted and managed and to identify additional issues that might need to be considered in relation to policymaking and practical solutions intended to facilitate secure data-intensive activities. Twelve scientists participated in this study. Our sample comprised three female and nine male respondents. The respondents had different professional and organizational backgrounds; see Table 1.

**Table 1:** Demographics of the scientists in the study

Profession	Organization	Gender
2 data managers of a bioinformatics infrastructure	6 from universities	9 males
1 medical scientist developing AI-based tools	2 from a national centre for molecular biosciences with a focus on health and environmental research	3 females
1 geneticist	2 from hospitals	
1 neuroscientist	1 from a county council	
2 epidemiologists	1 from a cancer research consortium	
1 nephrologist		
1 scientist in applied mathematics		
1 project coordinator		
1 legal adviser to research projects and a hospital		
1 data protection officer		

### Data collection

The interviews were performed between September 2019 and February 2020 by the first author (JVJ). First, we performed purposive sampling with criterion sampling, which means that the approached interview candidates met a particular criterion [22], and we focused on diverse professionals who were engaging in the collection, use and sharing of health data in Sweden. We consulted the contacts and webpages of different entities that collect, store, and transfer health data in Sweden (research groups, research infrastructures, and healthcare providers). Thereafter, we used snowball sampling to reach new interview candidates (n = 2) from specific areas. All of the candidates we approached were positive and eager to participate; they emphasized the pressing interview topic of health data handling. Two planned interviews had to be cancelled due to time constraints of the respondents (one changed jobs during that time, and another was not able to prioritize the interview due to work). The interviews lasted 29 to 72 min each and were conducted in Swedish, apart from one, which was conducted in English. The interviews were conducted in a quiet room, either at the first author’s workplace or in the interviewed expert’s office. One interview took place via Zoom due to the respondent being abroad. We began each interview by asking about the interviewee’s experience with using and sharing health data in their organization (e.g., what type of data do you collect, with whom is it shared, and for what purpose is it shared; are the data subjects able to give consent; which digital techniques are used; and what do you think about the future of your field). A semi-structured interview guide with open-ended questions [23, 24] was developed with the broader research team that this study was part of; see the Additional file 1.

A pilot interview was conducted with two colleagues to assess whether the questions stimulated reflection. Minor adjustments were made; for example, several of the questions were changed to probing questions and the order of the questions was changed.

### Analysis

The reordered interviews were transcribed verbatim in the spoken language of the respondents (11 Swedish and 1 English) by a professional transcription company. The transcripts were listened to in their entirety to verify the transcription. After all the transcripts were read again, meaning units (phrases, sentences, or paragraphs) through which ethical values and norms were explicitly or implicitly expressed were identified for further scrutiny. The material contained 86 meaning units in total. Atlas.ti software [25] and Microsoft Excel (2016) were used to assist in data management. During the next stage, we continued with a comparison of the meaning units, examining their similarities and differences from the perspective of ethical views. Open coding of each meaning unit was added, that summed up what being said in the text [26]. Each researcher performed this task separately, and then we jointly discussed our interpretations. Codes that reflected similar concepts were grouped, subcategories were formulated [26] and categories were connected to traditional ethical approaches; see Table 2. Thematic saturation was reached in relation to the primary aim of this data collection process. However, that was not a criterion for this study. When analysing the interviews in light of the primary aim, we found that the interviews contained detailed and rich descriptions of ethical approaches. Not reaching saturation would not have necessarily invalidated these qualitative findings; it may have simply meant that the “phenomenon had not yet been explored fully or sufficiently” [27].

**Table 2:** Example of the analytical process of the ethical approaches used by scientists who collect and share health data

Meaning units	Initial coding	Subcategory	Category
…and the patient has approved this and is informed that it will be shared and that it is understandable patient information, for example. The patient understands that it is voluntary and that they can say no as well	Give participants the opportunity to make an autonomous decision	Autonomy	Respect for rights (deontological approach)
…that is what people expect from us	Act in accordance with what is said	Keep promises	Respect for rights (deontological approach)
…the risk is that information will come out about people's diseases, which should not be in the public domain	Protect against bad consequences	Do no harm	Consideration of consequences (consequentialism)

### Ethical considerations

According to the Swedish Ethical Review Act, this study did not require ethical review, as it did not involve special categories of personal data. Nevertheless, all the procedures conducted in the study were in accordance with ethical conduct as described by Swedish law (SFS 2003: 460). E-mail invitations were sent to prospective candidates. These invitations included an overview of the project and the expected questions. After each expert agreed to participate, a time and a quiet interview location were agreed upon. We requested oral consent from all the respondents to record the interviews, transcribe them, and retain the transcripts for 10 years. When we requested their consent, the respondents were informed that they could withdraw from the study at any time with no explanation. Their names were replaced with codes, and only the first author had access to these data. All personal identifiers were removed so that the persons described were not identifiable, and care was taken so that the interview subjects could not be identified based on the details of their narratives.

## Results

The results describe the ethical approaches adopted by the participating scientists in relation to collecting, using, and sharing health data. In the analysis of this study, four main categories and fourteen subcategories were used to classify the discussions: 1) consideration of consequences (consequentialism), 2) respect for rights (deontological approach), 3) procedural compliance (procedural ethics), and 4) being professional (virtue ethics). An overview of the categories and subcategories is presented in Table 3. In the following, these categories and subcategories will be described and illustrated by quotes.

**Table 3:** The categories and subcategories of the ethical approaches attributed to the participating scientists in relation to sharing health data.

Category	Subcategory	No of respondents
Consideration of consequences (consequentialism)	Benefit to society	7
	Benefit to science	6
	Do no harm to individuals/non-maleficence	8
Respect for rights (deontological approach)	Right to a private sphere	6
	Autonomy	7
	Freedom	3
	Human dignity	2
	Keeping promises	5
	Justice	3
Procedural compliance (procedural ethics)	Effective approach	2
	Transparency towards data subjects and society	2
	Doing the right thing by default	2
Being professional (virtue ethics)	Being responsible	3
	Being respectful	2

To illustrate the weight placed on each ethical approach, the number of respondents who brought up the topic is mentioned. As is always the case in qualitative research, it is not possible to generalize on the basis of these numbers

### Category 1: consideration of consequences (consequentialism)

The consequentialist approach was expressed in three different ways: benefit to society, benefit to science, and do no harm to individuals/non-maleficence.

#### Benefit to society

The respondents argued that sharing individual health data benefits society in one way or another: data sharing may help explain the origin of diseases, be useful for the population due to the new development of treatments and save lives. Some respondents expressed that the more collaboration there is with academic scientific research and other academic projects or even with commercial companies developing new medical devices or drugs, the more benefit there will be to society. A concern was raised that excessively strict legal rules do not benefit society, as they may hinder beneficial research and technical development. The respondents pushed for open data sharing in the research community as a means to improve health care in the future.

Another consequentialist argument given for the wide reuse of health data was the need to maximize benefits to society by maximizing data reuse as much as possible. It was argued that people’s tax money should be used in a manner that is beneficial for the population. Because hospitals, universities, and national consortia in Sweden are financed with tax money, the data they collect or generate need to be widely used to maximize their benefits.

[…] when data are produced with tax money, for example, then they [tax payers] want them to be used as much as possible. There should be no obstacles to that, so that each [taxpayer] gets as much benefit as possible from the data that have been produced. They must then be shared with more scientists and so on. (Respondent 1, data manager)

#### Benefit to science

The respondents stressed that sharing data is not only beneficial for society but also a necessity in relation to answering certain research questions. Data sharing and reuse increase the possibility of making new discoveries. The respondents expressed the view that if the applicable legal rules are too strict, the law will hinder important research.

I understand that there are reasons for laws and stuff like that, but […] it is often a hindrance I think, being constrained in what you are allowed to do in this way. (Respondent 12, scientist in applied mathematics)

Since data collection is expensive, it was expressed that the research community needs to preserve existing data. Reuse is perceived as a way to make good use of pre-existing data. Even within this subcategory, two respondents expressed that they saw no great risk for harm. Rather, their view was that the good consequences for improved research outweigh the risk of people being harmed. They could imagine potential negative consequences, but they perceived this risk to be highly unlikely. Some of the respondents expressed enthusiasm about having access to data and the freedom to perform research. They were encouraged by these aspects and expressed a strong motivation to perform data-intensive research tasks.

Another aspect of sharing data responsibly is that science needs a good reputation to maintain trustworthiness. If the scientific sector is perceived as trustworthy, it has a good basis for participant recruitment and retainment. This aspect is also important in terms of maintaining people’s confidence in the research community for financial reasons since most research funding comes from taxpayers.

#### Do no harm to individuals/non-maleficence

The majority of the respondents acknowledged that there are threats to participants’ private spheres in the form of bad consequences if their personal health information falls into the wrong hands. They recognized that data can be lost and end up in the wrong hands; people can be identified, and data can be misused. People can sell these data or earn money by blackmailing individuals, threatening to disseminate their information about being in a risk group or having a certain disease. Insurance companies were mentioned as entities with an interest in this kind of information. Therefore, there is a risk that data ending up in the wrong hands can give people an economic disadvantage. Some of the respondents viewed this as very hypothetical, but they acknowledged that it could happen; therefore, protective measures must be taken to protect participants.

However, two respondents perceived the risk of individuals being harmed to be so small that it almost does not count. One respondent could not see how anyone could be interested in such participants’ health data.

I do not really think that there are very many who are super interested in these data in that way. (Respondent 7, epidemiologist)

People [scientists] are generally very unnecessarily anxious. People sit behind desks and behind paper and are very anxious about… ‘what if data comes out, and what if I do wrong and I do not know exactly what is right or wrong?’ There seems to be a bit of chaos, and GDPR has not exactly made things clearer or eased nervousness. My very personal attitude is that people are a little too anxious for their own health about this. (Respondent 4, project coordinator)

### Category 2: respect for rights (deontological approach)

In this category, the respondents described certain rights that need to be respected regardless of the consequences. The respondents explained that data subjects may be displeased and experience bad feelings if their information is in the hands of others. Within this category, we have included the respondents’ views on scientists’ right to perform research in the name of freedom of research.

#### Right to a private sphere

The respondents strongly emphasized people’s rights to be protected and not to be identified without consent. This was viewed as a matter of respecting other people and their integrity. The respondents expressed that if people’s health data are spread and fall into the wrong hands, their personal integrity is violated. Another opinion supporting this view was that personal data should not be freely accessible to everyone and that people have the right to decide what is known about them. One respondent thought that people are not sensitive to whether their personal information is known by random people but that if a neighbour or colleague knows the same information, it makes a difference. Hence, as it is difficult to determine a person’s level of acceptance to share information and level of vulnerability beforehand, the respondents expressed that actions and security levels need to be such that they meet the needs of the most vulnerable people.

So, in general, I think the risks are quite low, but it is more to protect personal integrity, … some care very much and do not want to give out their social security numbers or do not want to have such information everywhere, and you have to respect that. Then, there are others who do not care. (Respondent 6, epidemiologist)

#### Autonomy

The respondents explained that people are entitled to make decisions about sharing their health information according to their own wishes; autonomy is the core of participation in research. Participants have the right to decide for themselves whether or not their health data should be shared and with whom. Careful attention should be given to the information stage of research before data are collected so that potential participants are aware of the purpose of the research, the scientific method used, the reuse of their data, and expected result before consenting. It is essential for people to know whether their data will be used by others or for other purposes so that they are able to make an autonomous decision. Some of the respondents believed that the responsibility for the reuse of data lay in potential participants’ agreement. If participants consent to the potential risks and benefits of this process, then they should be trusted to understand what they are doing. People’s different preferences were also mentioned as a reason to leave the responsibility to decide to individuals. Moreover, giving them the opportunity to decide was seen as a way of respecting each participant as a person, the life that he or she has, and his or her experiences.

Who is responsible?… I would say the data subject. So, who is responsible for the data that is shared, yes, it is the individual, […] it is the individual who decides for him or herself. Do I want to accept this, that is, do I want to sign this consent? I want to buy a smart watch. I want a Google Home like this at home. Somewhere, consciously or unconsciously, individuals make a lot of choices in our society. So it must always start from the individual. […] Yes, and some people glide around in some form of ignorance as well. But I do not think that the government should be allowed to take responsibility for the fact that there happens to be ignorant individuals. (Respondent 4, project coordinator)

…and that the patient has agreed and is informed that it is shared and that it is understandable patient information, for example. The patient understands that it is voluntary and that they can say no as well. (Respondent 11, nephrologist)

#### Freedom

Moreover, the respondents voiced the importance of people being free to choose whether they want to participate in data collection not only to exercise their right to autonomy but also to realize the value of living a free life. It was argued that the state should not regulate everything and protect people from all possible incidents. That was perceived as an undesirable situation for our society.

…it is so dynamic [the technical development], so I feel very sceptical about appointing the state as responsible; I think it is the wrong way to go. One must be able to protect oneself in the first place. I am a little reluctant to leave it to a government to decide what kind of data I may share or what I allow someone else to do with it. (Respondent 4, project coordinator)

Two respondents made the opposite declaration, namely, that not all participants are autonomous. Therefore, they are not free to decide on their own because they are patients and dependent on care (which constitutes a power imbalance). Therefore, they need the protection of external rules that establish a more equal power balance alongside processes and guidelines that make the collection and use of people’s health data safe and rigorous. Thus, some are free thanks to the presence of rules.

Some other respondents focussed on freedom but form the perspective of data users (e.g., researchers) rather than data-subjects. The freedom to perform research was mentioned as an important norm of the research community. This norm is the basis of scientific research, and it needs to be protected; indeed, excessively strict and complicated rules hinder free research. Additionally, such rules were viewed as potential limitations on the level of knowledge that can be achieved, which goes against the goal of doing research.

#### Human dignity

Human dignity reflects the inherent worthiness of being a human. The respondents expressed the view that respect for human dignity may be less of a concern with the extensive data sets that scientists work with, since an individual becomes just one in a crowd. One reason for this phenomenon could be the social distance between a participant and the user of the corresponding data. The respondents emphasized that it is important to remember as a data collector that there are real people behind data; therefore, it is important to be careful when collecting, storing, and using data.

I think it is important that the scientists who use data and those who share data are aware that there are actually people attached to the information and that you should be very careful about how it is used and stored; there should not be any names and so on. (Respondent 6, epidemiologist)

#### Keeping promises

Keeping promises and following what has been agreed upon were mentioned in two ways. First, these concepts were described with the presupposition of an agreement being in place with informed consent and nothing hindering the use of people’s health data. If people agree to something, there is no need to question whether it is harmful or wrong, and rules are simply followed. Second, the respondents mentioned the importance of acting according to what has been promised. Indeed, things that have been said should be followed:

[…] that is what people expect from us. (Respondent 8, geneticist)

#### Justice

Another approach to having high standards in relation to control over participants’ health data that was expressed is that some people are in more vulnerable situations than others. Therefore, justice based on needs was expressed as a requirement when asking for people’s health data.

… there should be very high demands [on how we treat data]. I think there are some people who are weak and extra vulnerable. (Respondent 7, epidemiologist)

In contrast, the idea of justice was also deemed a motivator of openness of data in research and care. This argument is as follows: through open access to data, it is possible to represent all people in scientific research. In addition, a concern was raised that inequalities, which exist in the context of people’s health, will be maintained due to a lack of data representativeness. The concern is that in the long run, there will be an unequal distribution of care. Thus, greater openness in health data sharing will benefit us all, specifically underrepresented groups.

And that is a driving force of why it is so damn important that open sharing and equal sharing of data are as broad as possible […]it is because this group was not represented in the data on which this algorithm was practised… (Respondent 10, medical scientist developing AI-based tools)

On the other hand, entirely open access to data was viewed as problematic from the perspective that the professionals have put labour into the data collection and those who collect and analyse data should be recognized for their accomplishments. Some of the respondents noted the importance of scientists obtaining credit for the work they perform before their data are shared.

You have to get credit for what you have done, but you can still share it with others. (Respondent 3, neuroscientist)

### Category 3: Procedural compliance (procedural ethics)

In addition to selecting actions based on what they perceive to be the good and right thing to do, participants focus on the process. Many respondents voiced the need to have good data collection and sharing procedures to ensure professional behaviour. They expressed a desire to do the right thing, but they wanted to decrease the obstacles that hold back beneficial development and make administrative work inefficient. They expressed a desire for a data-sharing routine delineating correct actions; it should be simple to follow rules and not think all the time about what is right or wrong. For example, there should be a practical computer system so that data files do not have to be e-mailed to others and a simple ethical approval system that is comprehensible. Rules implemented to facilitate good procedures were perceived as giving freedom to research and innovate.

[…]but I have gotten emails with social security numbers and addresses for people; this should not happen. I do not want that in my mailbox. (Respondent 6, epidemiologist)

As a new scientist, I think you become overwhelmed, and then I think that many start to cheat. Many do not apply [for ethical approval], but they run their race. (Respondent 8, geneticist)

However, one respondent emphasized that rules need to be flexible to adapt to changing circumstances (e.g., technological development and new research questions). These changes can be difficult to foresee; therefore, the need to be transparent was viewed as important in a changing world. Being transparent with the community and with respondents was also viewed as a good path for an ethically sustainable research environment.

So, this is a balancing act; they [the regulations and processes] must be alive... the ethical regulations must be alive so that they can adapt, because if there is new technology that makes it possible to do something we cannot do today, we must be able to say, you cannot do it this way; you have to do it this way or protect the individual in some way. (Respondent 8, geneticist)

### Category 4: being professional (virtue ethics)

Finally, our analysis revealed expressions of being professional and how one should be as a scientist. This dimension was mostly related to the character that data collectors and users have or should have. One view was that scientists (data collectors) are not interested in individuals’ health statuses and do not dissects data on an individual level; therefore, the risk of something going wrong is negligible.

Scientists reported a need to be respectful and responsible, which are considered important virtues of their professions. However, some of the respondents believed that data users can be affected by their interest in discovery and forget or stretch the rules because they are curious.

[…] and then these people start to fumble a bit and hand data over to some company they collaborate with, which they think is very exciting, and so it starts to slide. (Respondent 4, project coordinator)

## Discussion

The aim of this study was to describe the ethical approaches adopted by scientists who collect, use and share individual-level health data with new users. The scientists that participated in the present study referred to and described norms and values in the context of their practical work related to handling people’s health data. The category ‘consideration of consequences’ and its subcategories (benefit to society, benefit to science, and do no harm to individuals/non-maleficence) and the category ‘respect for rights’ with its subcategories (right to a private sphere and autonomy) were represented by all the respondents, accounting for the majority of the interview material. The subcategories of ‘respect for rights,’ namely, freedom, human dignity, keeping promises, and justice, were not as prominently represented among all the respondents. Following these categories were different approaches to the work of data handling. First, the respondents discussed the need for good procedures to support professionally ethical behaviour. The need for good procedures was described with strong emotional expressions (e.g., irritated or frustrated), both from the perspective of the safety of data subjects and from that of ensuring a good working environment. Second, the final category of ‘professional conduct’ was not mentioned by all the respondents, but it was very prominently discussed by a few. This could be further explored.

When considering the contributions of all the respondents, we observed differences between the various ethical approaches. We observed internal contradictions between doing good for society and individuals’ right to decide for themselves. The EU General Data Protection Regulation 2016/679 (GDPR) strengthens individuals’ rights regarding their personal data [28]. The GDPR embodies the principle of informational self-determination by increasing transparency requirements for data collection practices. The respondents who participated in this study focused heavily on the fact that the result of data collection should benefit society and on informed consent, two basic requirements for personal data processing according to the GDPR. The respondents’ ethical approaches in this study are in accordance with values expressed by data subjects in earlier studies. Data subjects have expressed that participants should be able to decide with whom data are shared, what types of data are shared, for what purposes data are shared, and consequently what types of risk they are willing to take [16, 29]. This is in line with the result from this study. However, some of the respondents in this study seem to place much responsibility on individual data subjects, disregarding the power imbalance between a data subject and the individual collecting and processing his or her data. Some of our respondents thought that relying solely on a data subject’s consent provides insufficient protection. Consent is still necessary, but additional safeguards need to be in place [30]. Thus, the GDPR points to further safeguards, for example, encryption and pseudonymization. Furthermore, some of the respondents expressed a societal obligation to use data to the greatest extent possible for the benefit of science and society, particularly when data generation is publicly funded. We can identify a risk from this perspective, namely, that data users might prioritize benefits to science and society above individuals’ fundamental rights, as they want to maximize taxpayers’ financial contributions for the benefit of the health field. Indeed, some consider the risks to the individual so small that they are negligible and outweighed by the benefits that result from data processing. They seemingly disregard that in today’s data-driven society, risks to individuals no longer just comprise the potential for physical harm but also include that for informational harm, which occurs when information about a person falls into the wrong hands, resulting in a risk of harm (e.g., discrimination and stigmatization).

There were mixed views regarding whether subjects’ data are of interest to people other than those collecting and using these data. Some of the respondents felt that there is a real threat that data can fall in the wrong hands and thereby harm people. Some of the respondents in this study could not fathom others being interested in these data. They perceived the risk as being so low that it could be ignored. In the academic risk literature, a risk that is so low that it can be ignored is called *de minimis* [31]. This is also a legal term meaning that insignificant matters do not need to be considered – “the law does not concern itself with trifles” [32]. From an ethical point of view, one should not ignore small risks or treat them very differently from other risks. Lundgren and Stefánsson [33] argue that there “is no probability threshold below which risks can rationally be treated categorically differently from other risks.” Furthermore, it is well established that subjects’ health data can be misused or processed for unintended purposes [34, 35]. Moreover, on an individual level, the consequences of a small risk can be substantial and thus inflate the risk. Health data have been compared to a *new oil* due to their lucrative potential [36]; therefore, they might attract fraudsters. Another comparison made in healthcare is that health data are the *new blood*. The proposed comparison is that health data are “digital specimens and should be treated with the same rigour, care, and caution afforded to physical medical specimens” [6]. The harm resulting from their misuse is not only physical but also psychological and emotional [37]. One ongoing debate is whether a patient’s digital twin should be viewed as an extension of his or her body [38, 39]. Even if a digital twin is only a reduction of the corresponding person and cannot be considered autonomous, its integrity should be respected with regard to how the relevant data are used, shared, and secured. The reason for this, as mentioned earlier, is that data that are not protected adequately could attract fraudsters.

Moreover, these risks increase as interest in big data – and the number of new commercial actors active in its analysis and use – increases. Indeed, interest in accessibility to data has increased greatly over the last few years [40–42]. The reason for this increased interest in large amounts of health data is the need, for example, to have training sets for the development of AI technologies [43, 44]. The data economy is blossoming, with start-up companies looking to earn money from data-based innovations [45]. The greatest threat to the appropriate use of data is perhaps not primarily hackers and other criminals but companies that have commercial profit as their top priority. This means that reidentification is not the only risk at stake; indeed, respect for the preferences of individuals is another value in question. While reidentification can be prevented through technical means (pseudonmization, encryption, etc.), individuals’ preferences are not respected by default. They need to be taken in to account from the beginning.

However, as the respondents of this study expressed (subcategory: benefit to society), overprotection can also lead to harm. It is important to protect people’s individual-level health data, but not to the extent that obstacles for new inventions and better health care based on big data, machine learning, and learning health systems are formed [6]. Advances in information technology are challenging the traditional view of consent and anonymization as primary safeguards, showing that privacy-enhancing technologies can contribute considerably to protecting people’s health data. This is a fine balance that requires interdisciplinary collaboration. In this context, protecting people’s health data while providing flexible and efficient technical solutions is a challenge. By focusing on the FAIR principles of findability, accessibility, interoperability, and reusability, we can point the way forwards in terms of facilitating data sharing more systematically [46]. However, certain methodological and organizational challenges remain. The FAIR principles only call for the explication of access conditions without specifying how data sharing should be facilitated or organized. These principles can provide guidance on how to think about all the “design choices embodied in data, developing new modes for respecting participants’ rights, and coming up with robust measures for valuing data sharing which do not reproduce the problems related to current scientific reward systems” [47].

Privacy is traditionally defined as the right to be left alone [48]. However, different contexts give rise to different expectations and preferences related to privacy [49]. Contextual rules about how information should flow depend on the actors involved, the accessibility of data, and the purpose of data access. Nissenbaum explains that privacy is violated when contextual rules are contravened [49]. Responses regarding why such violations are wrong can traditionally be divided into two categories – consequentialist and deontological concerns [6]. This was in line with our respondents’ thoughts. Consequentialist concerns relate to all the possible bad things that can happen and individuals’ uncomfortable feelings of being observed or of losing control, regardless of whether there is an actual threat. Deontological concerns do not depend on whether negative consequences are experienced. When a data breach occurs, privacy has been violated even if no one uses an impacted person’s information against him or her or if the person never even becomes aware that the breach occurred.

In addition to expressing consequentialist and deontological concerns, the results of this study highlight procedural ethics [50] as an important ethical approach with respect to the health data landscape. Having a robust and harmonized procedure for sharing data not only protects individuals but also assures scientists that they are acting ethically and are legally compliant in relation to data subjects. Procedural ethics encompasses processes such as the development of a data management plan and the establishment of a framework for ethical behaviour. Day-to-day practices need to be established such that data collectors and users follow the rules and the ethical concerns expressed by people and the law. An example of this is the GDPR rules regarding data protection by design and by default, which aim to build legal rules into technology and make legal compliance and privacy preservation the default technical option [51–53]. The notion of ethics by design has sprung out of this and similarly seeks to embed ethical principles into technological design and development [54]. Everyday procedures should be established in a similar fashion to ensure legal and ethical compliance by default, ease the administrative burden of data users and benefit data subjects.

Ethical and legal issues when sharing health data have grown in tandem with advances in digital health and computing technologies. This study combines empirical investigation with ethical reflection. Empirical investigations of the ethical approaches used by scientists can identify relevant moral issues and describe scientists’ beliefs and attitudes, which can be relevant for the ethical issue of sharing health data digitally. Empirical ethics can improve the context-sensitivity of ethical deliberation [19]. Some of the respondents of this study stressed that rules and ethical concerns need to be more thoroughly integrated into practice. The lack of a harmonized system and complex processes hinder beneficial technological development and data subjects’ security. We believe that certain ethical obstacles need to shift from ethical issues to ‘law concepts.’ There is a risk that ethics becomes a sort of replica of law or a softer version of law (55). Therefore, we suggest that the time is now ripe to move from policy discussions to practical solutions based on principles. Our respondents expressed a desire to do the right thing, but practical reality is sometimes a hindrance.

## Conclusion

These empirical findings suggest a need for practical procedures that make it easier for data collectors and sharers to follow ethical principles and laws regarding data sharing. We suggest that it is an opportune time to move on from policy discussions to practical technological solutions based on principles. Data collectors need better technical and practical guidance to follow ethical and legal demands. Scientists have expressed that uncertainties in the application of rules divert their time and resources from what they are trained to do – creating new treatments and finding new diagnostic tools.

## Supplementary Information

**Additional file 1. **Governance of health data in cyberspace  - Expert interview guide. The interview guide for the study

## Data Availability

The interview data sets that were generated and analysed during the current study are not publicly available because the participating individuals could be identified, and consent for making their data available was not obtained. However, they can be made partly available by the corresponding author upon reasonable request.

## Abbreviation

GDPR: General data protection regulation
