# Supplementary material for: What ethical approaches are used by scientists when sharing health data? An interview study
Source: BMC Med Ethics. 2022 Apr 11;23:41. doi: 10.1186/s12910-022-00779-8 (PMC9004072; doi:10.1186/s12910-022-00779-8)
Supplement: Supplementary file 1 — Additional file 1. Governance of health data in cyberspace - Expert interview guide. The interview guide for the study [file 12910_2022_779_MOESM1_ESM.docx]

**Governance of health data in cyberspace – Expert interview guide**

**Introduction**

Thank you all for taking the time to attend this interview today.

My name is xxx, and I work for the xxx.

We are conducting a research project called the Governance of Health Data in Cyberspace, which is being funded by NORDFORSK and the ESRC. As you know, over the last 20 years, healthcare and medical research has become increasingly dependent on the digital collection and sharing of health information (e.g., biomedical data, diagnoses, treatments, and genomic sequencing). Technological developments are moving very fast, allowing the data collected in one context to be used for multiple purposes.

The aims of this project are twofold: 1) to understand how citizens would like the secondary use of their health-relevant data to be governed and to identify appropriate governance solutions; and 2) to assess how users of health and other related data use and implement governance strategies and what barriers and facilitators may exist in relation to these in the context of emerging technologies that harness people’s data from a variety of sources. Therefore, we are conducting a series of focus groups and individual interviews with members of the public and with experts to explore these aims, and the findings will help inform a further study intended to elicit citizen preferences regarding governance called the Discrete Choice Experiment, which will be conducted in Iceland, Sweden, Norway and the UK.

Our citizen focus groups are exploring the circumstances under which individuals judge the sharing of their health-relevant information to be inappropriate.

We have invited you as an expert in the area of health data sharing to contribute to our understanding of how data sharing is conducted and managed and identify additional issues that we might need to consider.

The findings from this interview will be analysed, summarized, and later written up for publication in peer-reviewed journals. The results will also inform the development of a survey designed to measure people’s preferences regarding digital health data sharing governance.

We will begin with some introductory questions about health data sharing and use, and then I will ask you to consider and share your thoughts about that topic.

Please note that there are no right or wrong answers. It is important to emphasize that your participation is voluntary; it is possible to withdraw from the study at any time, even during the discussion or after the discussion has taken place. Please note that this discussion will be audio recorded and will later be transcribed by a transcription firm.

Are there any questions before we begin the interview?

**Questions**

*Opening questions – warm up round (15 minutes)*

1a. Can you start by telling me what you do that involves data collection and the use and sharing of individual-level health data?

Prompt: In regard to the sharing of health-related data in your area of work, can you give me examples of the types of data that might be shared and for what purposes they are shared? With whom do you share these data? How do you think this might change in the next 5-10 years? What digital technologies do you use?

1b. What are the important factors you consider when health data are being shared and used?

1c. Can you describe the risks and benefits experienced by data subjects when their health data are shared for different purposes and with third parties? How do you consider the risks when making decisions to share data?

*Governance and oversight of data sharing (20 minutes)*

2a. What approvals, processes and guidelines are employed for secondary uses of data in your area of work?

2b. In your opinion, how do these affect your area of work? If applicable, what about future practice?

*Barriers and facilitators in governing health data sharing (30 minutes)*

I would now like to ask you some questions about the application or implementation and use of different oversight or governance mechanisms you have mentioned.

3a. Are the current governance and oversight mechanisms we talked about sufficient for protecting data subjects, or is there more to be done? From a user perspective, are these well delineated?

3b. Are there any oversight or governance mechanisms that need to be better developed and adopted more widely?

3c. In your opinion, who should be responsible and accountable for the sharing of digital health data?

*Closing questions (5 minutes)*

4a. We have now discussed your perspective on health-relevant data sharing; are there any final thoughts?

4b. Is there anything we haven’t discussed that you would like to mention?

**Thank you for your contributions today.**
